# Supplementary material for: Droplet digital PCR-based testing for donor-derived cell-free DNA in transplanted patients as noninvasive marker of allograft health: Methodological aspects
Source: PLoS One. 2023 Feb 24;18(2):e0282332. doi: 10.1371/journal.pone.0282332 (PMC9955980; doi:10.1371/journal.pone.0282332)
Supplement: S1 Text — (DOCX) [file pone.0282332.s001.docx]

**S1 Text. SNP assay design criteria**

SNP assay design was done in various steps: Identification of candidate SNP at National Center of Biotechnology Information (NCBI), search for common SNPs around the SNP of interest, selection and test of primer and probe candidates in Primer3, and finally a BLAST and a final PCR product check.

Candidate SNPs were selected from NCBI [variation viewer]. Source database: dbSNP / ClinVar: Yes / Most severe clinical significance: Benign / Variant type: Single Nucleotide variant / 1000 Genomes MAF: ≥ 0.5 / ExAC MAF: ≥ 0.1 / Has publications: Yes.

Rs numbers with MAF 0.4-0.5 were selected.

Search for common SNPs (>1%) was done using the Genome Browser from the University of California, Santa Cruz (UCSC). No additional common SNPs >1% were accepted within 100 bp from the SNP.

DNA sequences were then imported into Primer3 (0.4.0) to primers and probes.

Primer criteria: Size: 18-25 bp / Tm: 60-65°C / GC-%: 40-60 % / product size range: 60-90 bp

Probe criteria: Size: 15-30 bp / Tm: 60-75°C / GC-%: 20-80 %

Additional criteria were manually applied Primer3-generated candidates:

1. Avoid repetitions of three and more G and C in the primer sequences
2. See to that there is a G or C at the 3’ end of the primer sequences
3. Avoid four G repeats in the probe sequence
4. Avoid G at the 5’ end of the probe
5. Avoid overlap between primers and probes

Selected assay candidates were then assessed using BLAST, selecting assays with no additional amplification sites (or predicted product amplification).

A final check was done using the PCR tool at UCSC, to check the number of predicted products for selected primer pairs. If an additional product was predicted, the assay was discarded.

SNP assays was then selected, representing various chromosomes.
