# Supplementary figures and images for: Droplet digital PCR-based testing for donor-derived cell-free DNA in transplanted patients as noninvasive marker of allograft health: Methodological aspects
Source: PLoS One. 2023 Feb 24;18(2):e0282332. doi: 10.1371/journal.pone.0282332 (PMC9955980; doi:10.1371/journal.pone.0282332)

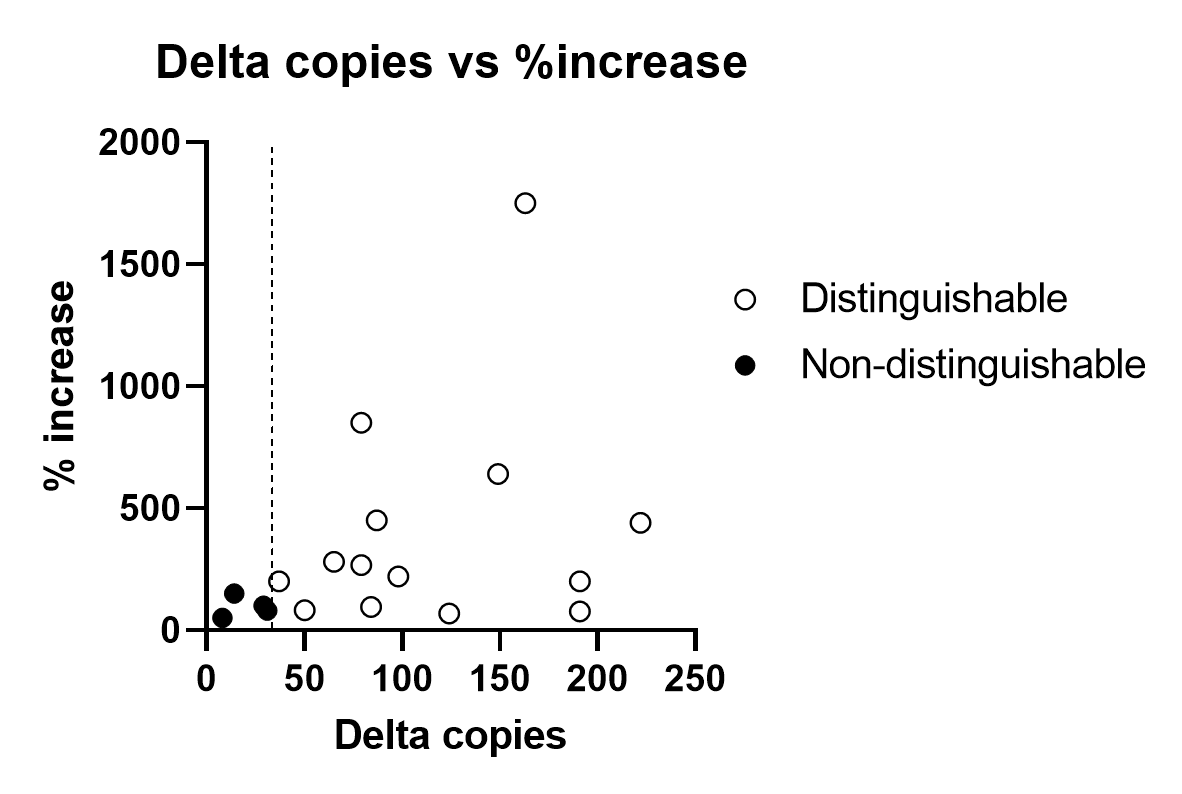

Supplement: S1 Fig — Delta copies of data from Spike 3–5 plotted against percentage increase of fraction shown to mark a difference (dashed line of 37 copies per reaction) between distinguishable and non-distinguishable data. (TIF) [file pone.0282332.s002.tif]
