# Supplementary material for: Droplet digital PCR-based testing for donor-derived cell-free DNA in transplanted patients as noninvasive marker of allograft health: Methodological aspects
Source: PLoS One. 2023 Feb 24;18(2):e0282332. doi: 10.1371/journal.pone.0282332 (PMC9955980; doi:10.1371/journal.pone.0282332)
Supplement: Attachment — Submitted filename: Response to Reviewers.pdf [file pone.0282332.s003.pdf]

Rigshospitalet

The Centre of Diagnostic Investigations

Department of Clinical Immunology

Blegdamsvej 9
DK-2100 KBH Ø

Section 2034
Direct 61668141

PLOS ONE

Date: 8 February 2023

Response to Reviewers

I thank the editor and the reviewer for their work and positive comments regarding our manuscript. It is highly appreciated. A new and revised manuscript has been uploaded, with and without track changes as requested by the editor.

Regarding Journal Requirements:

Ad 1) I have ensured to the best of my knowledge that the manuscript meets the PLOS ONE's style requirements, which includes a change in the manuscript reference style for supplementary information. The specific reference reading "…in supporting information S1 Text." was changed to "…in S1 Text." (Page 7). I hope this is now correct.

Ad 2) I have matched the information provided in the 'Funding Information' and 'Financial Disclosure'. (I understand 'Financial Disclosure' as the comment of financial interest which is stated in the Cover Letter). Consequently, the amended comment in the new Cover Letter is now identical to the information given under 'Funding Disclosure'. In addition, I have provided grant numbers for each grant, as requested.

Ad 3) An ethics statement was added as the first section under Methods (page 5 and 6). It reads as follows:
"This study was a quality assurance project using blood samples from healthy volunteers. Informed and written consent was obtained from all participating volunteers when blood samples were collected. According to Danish law, no ethical approval was required, because this was a quality assurance project, thus waiving the need for ethics committee ap-

proval of the study. No DNA information related to disease was examined, and only SNPs with no known clinical significance were used, thus avoiding the challenges of reporting incidental findings."

Regarding additional Editor Comments:

On the issue of including a small validation of clinical samples, I agree that one would often expect to see an analytical validation accompanied by a clinical validation. And adding a few clinical samples might seem like a good idea and a natural step towards a clinical validation. We did exactly that in our first study on cell-free DNA, almost 20 years ago [PMID: 16231312]. However, this field is far more complicated. Thus, for a number of key reasons, as listed below, adding a small number of clinical samples will not improve our manuscript. And we therefore choose not to.

1. *A true clinical validation is much larger than often realized.* A clinical validation in the context of transplantation patients is complicated. Importantly, no actual clinical validation has yet been done in this field. Such validation must include a) monitoring several patients over several years just to demonstrate correlation; b) a potential threshold must be identified and tested as the indication for action and medical intervention; c) a threshold-based guidance of medical intervention must then be tested prospectively to demonstrate clinical value. We mention these important issues in our manuscript. A clinical validation is thus unfeasible at this time. Adding a small validation should not be regarded as a clinical validation.

2. *A small number of clinical samples is unnecessary*. The only reason that you would add a number of clinical samples to a methodological study is when your mimicking material is vastly different from the clinical material. For example, several studies have used artificial DNA samples and then clinical plasma samples to see if the presented assays would work in plasma as well. We used plasma samples obtained and processed under the same clinical conditions as expected for clinical plasma samples. The cfDNA in plasma is similar. Similar in fragmentation and size. We discuss these issues in the manuscript. Thus, adding a small validation would add no real value.

3. *Meticulous presentation of the methodological aspects is crucial*. In transplantation, cfDNA testing is a young science, and it is difficult to convey important methodologically details to clinicians and medical scientists. Therefore, special attention to methodological aspects is needed and warrants meticulous presentation in the literature. The issue of threshold in kidney testing is a good example of confusion related to methodological aspects and insufficient validation. No precise threshold has been identified to guide medical intervention; however, many clinicians believe that such thresholds exist—even though the most recent paper clearly shows otherwise [PMID: 34953773]. We discuss these issues in the manuscript. Important methodological issues are thus not always sufficiently dealt with. Adding a small clinical validation may only add further confusion about assay readiness. By contrast, a clear separation between analytical and clinical validation is necessary and desirable in my opinion. Focus should be on the methodological aspects at first.

4. *Timely publication of method is highly important*. As discussed under point 1, a clinical validation requires several steps and monitoring of patients over several years. Therefore, it is important to present the method for other scientists quickly in the literature to help others start similar endeavors to save patient lives. The ddPCR assay we present is an older method (taken from the literature) that we have improved significantly. We could only re-use half of the assays from the old method and thus designed new assays. This is a very important message to get out quickly, so that other scientists will not waste unnecessary resources trying to implement the old setup. As such, the key information in our manuscript is to present reliable SNP assays which can be used for dd-cfDNA testing in plasma.

5. In addition, *ethical rules restrict us from ad hoc testing a small number of samples*. Ethics committee rules imply that we cannot just add a small group of samples. To test clinical samples, such samples must be part of a clinical project, in which a sufficient sample size must be calculated and accounted for, including an accepted and funded feasibility plan to obtain clinical goals. GDPR rules prevent us from just adding a small group of samples, if tested outside an approved clinical study. So, even if we agreed that this was the correct way forward, we would not be allowed to do so.

Based on these five key arguments, we abstain from adding clinical samples. Rather, we adhere to the rationale of presenting methodologically aspects timely, and then present thorough clinical data in years to come. Thus, we now focus our resources on planning different clinical projects in which we will apply this method. In the meantime, it is important to publish this method and our meticulous description.
Thank you for your patience reading our arguments for this position.
I hope the editor will approve of these considerations.

And I thank again the reviewer for the reviewer’s highly positive feedback.

Yours sincerely,

Frederik Banch Clausen
DMSc, PhD
Senior Research Scientist
Copenhagen University Hospital
Denmark
Frederik.Banch.Clausen@regionh.dk
