# Supplementary material for: Droplet digital PCR-based testing for donor-derived cell-free DNA in transplanted patients as noninvasive marker of allograft health: Methodological aspects
Source: PLoS One. 2023 Feb 24;18(2):e0282332. doi: 10.1371/journal.pone.0282332 (PMC9955980; doi:10.1371/journal.pone.0282332)
Supplement: Attachment — Submitted filename: Response to Reviewers.docx [file pone.0282332.s004.docx]

**Response to Reviewers**

I thank again the editor for the editor’s work on improving our manuscript.

Regarding Journal Requirements

I have reviewed all the references. None of them cite papers that have been retracted.

However, references 40 and 45 do not appear at PubMed.

Reference 40 is an abstract, and it is still available and can be found on google scholar.

Reference 45 is a white paper from a commercial company, and it can also be found on google scholar. As all cited papers are available, I have made no amendments to the reference list.

Regarding Additional Editor Comments
This issue with the format of the result section is based on a simple mistake.

The fig titles are not sub-sections of the result section (the result section has no sub-sections). It is the caption and legend of each figure, inserted into the text after the paragraph where the figure is first mentioned. This has been done according to the guidelines from PLOS ONE. Specifically, the guidelines state: “Figure captions are inserted immediately after the first paragraph in which the figure is cited.” Elsewhere it says: “The caption may also include a legend as needed.” Then, one can download an example (Download sample manuscript body), in which the fig caption and legend is presented exactly as I have done it in the manuscript. Thus, I have followed the journal’s instructions meticulously. I do agree that this insertion may be confusing. To alleviate the confusion, I have added the following sentence on top of each fig paragraph: “[The paragraph below is Fig 1’s caption and legend]” (writing Fig 1’s for Fig 1 and Fig 2’s for Fig 2 and so forth). In this way, one should deduce that the paragraph is not a part of the text of the result section.

I hope this is helpful, although it is a deviation from the guidelines.
